# Emotion Assessment Using Feature Fusion and Decision Fusion Classification Based on Physiological Data: Are We There Yet?

**DOI:** 10.3390/s20174723

**Published:** 2020-08-21

**Authors:** Patrícia Bota, Chen Wang, Ana Fred, Hugo Silva

**Affiliations:** 1 Instituto Superior Técnico (IST), Department of Bioengineering (DBE) and Instituto de Telecomunicações (IT), Av. Rovisco Pais n. 1, Torre Norte-Piso 10, 1049-001 Lisbon, Portugal; afred@lx.it.pt (A.F.); hsilva@lx.it.pt (H.S.); 2 State Key Laboratory of Media Convergence Production Technology and Systems, Xinhua News Agency & Future Media Convergence Institute (FMCI), Xinhua Net, Jinxuan Building, No. 129 Xuanwumen West Street, Beijing 100031, China; wangchen@news.cn

**Keywords:** emotion recognition, physiological signals, machine learning, signal processing

## Abstract

Emotion recognition based on physiological data classification has been a topic of increasingly growing interest for more than a decade. However, there is a lack of systematic analysis in literature regarding the selection of classifiers to use, sensor modalities, features and range of expected accuracy, just to name a few limitations. In this work, we evaluate emotion in terms of low/high arousal and valence classification through Supervised Learning (SL), Decision Fusion (DF) and Feature Fusion (FF) techniques using multimodal physiological data, namely, Electrocardiography (ECG), Electrodermal Activity (EDA), Respiration (RESP), or Blood Volume Pulse (BVP). The main contribution of our work is a systematic study across five public datasets commonly used in the Emotion Recognition (ER) state-of-the-art, namely: (1) Classification performance analysis of ER benchmarking datasets in the arousal/valence space; (2) Summarising the ranges of the classification accuracy reported across the existing literature; (3) Characterising the results for diverse classifiers, sensor modalities and feature set combinations for ER using accuracy and F1-score; (4) Exploration of an extended feature set for each modality; (5) Systematic analysis of multimodal classification in DF and FF approaches. The experimental results showed that FF is the most competitive technique in terms of classification accuracy and computational complexity. We obtain superior or comparable results to those reported in the state-of-the-art for the selected datasets.

## 1. Introduction

Emotion is an integral part of human behaviour, exerting a powerful influence in mechanisms such as perception, attention, decision making and learning. Indeed, what humans tend to notice and memorise are usually not monotonous, commonplace events but the ones that evoke feelings of joy, sorrow, pleasure, or pain [1]. Therefore, understanding emotional states is crucial to understand human behaviour, cognition and decision making. The computer science field dedicated to the study of emotions is denoted as Affective Computing, whose modern potential applications include, among many others: (1) automated driver assistance—e.g., through an alert system monitoring and warning the user for sleepiness, unconscious or unhealthy states potentially hindering driving; (2) healthcare—e.g., through wellness monitoring applications identifying causes of stress, anxiety, depression or chronic diseases; (3) adaptive learning—e.g., through a teaching application able to adjust the content delivery rate and number of iterations according to the user enthusiasm and frustration level; (4) recommendation systems—e.g., assisting and asserting personalised content according to the user preferences as perceived by their response.

Emotions are communicated via external (facial or body expressions such as a smile, tense shoulders, and others) and internal body expressions (alterations in heart rate (HR), respiration rate, perspiration, and others). Such manifestations generally occur naturally and subconsciously, and their sentic modulation can be used to infer the subjects’ current emotional state. Acquired in a systematic daily setting, it could be possible to infer the probability of a subjects’ mood for the following day and their health condition.

External physical manifestations (e.g., facial expressions) are easily collected through a camera; however, they present low reliability since they depend highly on the user environment (if he is alone or in a group setting), or cultural background (if the subject grew up in a society promoting the externalisation or internalisation of emotion), and can be easily faked or manipulated according to the subject goals, compromising the assessment of the true emotional state [2]. On the other hand, for internal physical manifestations, these constraints are less prominent, since the subject has little control over his bodily states. Alterations in the physiological signals are not easily controlled by the subject, thus, these entitle a more authentic insight into the subject emotional experience.

Given these considerations, our work aims to perform a comprehensive study on automatic emotion recognition using physiological data, namely from Electrocardiography (ECG), Electrodermal Activity (EDA), Respiration (RESP), Blood Volume Pulse (BVP) sensors. This choice of modalities is due to three factors: (1) Data can be easily extracted from pervasive, discrete wearable technology, rather than more intrusive sensors (e.g., Electroencephalography (EEG), or Functional near-infrared spectroscopy (fNIRS)); (2) Widely reported in the recent state-of-the-art; (3) Publicly available multimodal datasets validated in literature. We use five public state-of-the-art datasets to evaluate two major techniques: Feature Fusion (FF) versus Decision Fusion (DF) on a feature-based representation, exploring also an extensive set of features comparatively to previous work. Furthermore, instead of the discrete model, the users’ emotional response is assessed on the two-dimensional space: Valence (measuring how unpleasant or pleasant is the emotion), and Arousal (measuring the emotion intensity level).

The remaining of this paper is organised as follows: Section 2 presents a brief literature review on ER, with special emphasis on articles that describe the datasets used in our work. Section 3 describes the overall machine learning pipeline of the proposed methods. Section 4 evaluates our methodology in five public datasets. Lastly, in Section 5, the main conclusions of this work are presented along with future work directions.

## 2. State of the Art

In literature, human emotion processing is generally described using two models: One decomposing emotion in discrete categories divided into basic/primary (arriving from innate, fast and in response to “flight-or-fight” behaviour) and complex/secondary emotions (deriving from cognitive processes) [3,4]. On the other hand, the second model quantifies emotions into continuous dimensions. A popular model, proposed by Lang [5], suggested a Valence (unpleasant–pleasant level) versus Arousal (activation level) two-dimensional model [6], which we adopt in this work. Concerning affect elicitation, it is generally performed through films snippets [6], virtual reality [7], music [8], recall [9], or stressful environments [6], with no commonly established norm on which is the optimal methodology for ER elicitation.

Regarding the automated recognition of emotional states, it is usually performed based on two methodologies [2,10,11]: (1) Traditional Machine Learning (ML) techniques [12,13,14]; (2) Deep learning approaches [15,16,17]. Due to the limited size of existing datasets, most of the work focuses on traditional ML algorithms, in particular Supervised Learning (SL), such as Support Vector Machines (SVM) [18,19,20], k-Nearest Neighbour (kNN) [21,22,23], Decision Trees (DT) [24,25], and others [26,27], with the SVM method being the most commonly applied algorithm, showing overall good results and low computational complexity.

Many physiological modalities and features have been evaluated for ER, namely Electroencephalography (EEG) [28,29,30], Electrocardiography (ECG) [31,32,33], Electrodermal Activity (EDA) [34,35,36], Respiration (RESP) [26], Blood Volume Pulse (BVP) [26,35] and Temperature (TEMP) [26]. Multi-modal approaches have prevailed; however, there is still no clear evidence of which feature combinations and physiological signals are the most relevant. The literature has shown that the classification performance improves with the simultaneous exploitation of different signal modalities [2,8,10,37], and that modality fusion can be performed at two main levels: FF [24,38,39] and DF [8,26,37,40,41]. In the former, features are extracted from each modality and latter concatenated to form a single feature vector space, to be used as input for the ML model. On the other hand, in DF, from each modality, a feature vector is extracted to form a classifier prediction through a voting system. Hence, with *k* modalities, *k* classifiers will be created leading to *k* predictions that can be combined to yield a final result. Both methodologies are found in the state-of-the art [42], but it is unclear which is the best to use in the area of ER using multimodal physiological data obtained from non-intrusive wearable technology.

Detailed information on the current state-of-the-art in a more generalized perspective, we refer the reader to the surveys [2,11,43,44,45,46,47] and references therein, where a comprehensive review of the latest work on ER using ML and physiological signals can be found, highlighting the main achievements, challenges, take-home messages, and possible future opportunities.

The present work extends the state-of-the-art of ER through: (1) Classification performance analysis, in the arousal/valence space, of ER for five publicly available datasets that cover multiple elicitation methods; (2) Summarising the ranges of the classification accuracy reported across the existing literature for the evaluated datasets; (3) Characterising the results for diverse classifiers, sensor modalities and feature set combinations for ER using accuracy and F1-score as evaluation metrics (the later not being commonly reported albeit important to evaluate classification bias); (4) Exploration of an extended feature set for each modality, analyzing also their relevance through feature selection; (5) Systematic analysis of multimodal classification in DF and FF approaches, with superior or comparable results to those reported in the state-of-the-art for the selected datasets.

## 3. Methods

To evaluate the classification accuracy in ER from physiological signals, we adopted the two dimensional Valence/Arousal space. As previously mentioned, the ECG, RESP, EDA, and BVP signals are used, and we compare FF and DF techniques in a feature space based framework. In the forthcoming sub-sections, a more detailed description of each approach is presented.

### 3.1. Feature Fusion

As previously mentioned, when working with multi-modal approaches the exploitation of the different signal modalities can be performed resorting to different techniques. We start by testing the FF technique. In FF, the features are independently extracted from each sensor modality (in our case ECG, BVP, EDA, and RESP), and are concatenated afterwards to form a single, global, feature vector (570 features for EDA, 373 for ECG, 322 for BVP, and 487 for RESP, implemented and detailed in the BioSPPy software library https://github.com/PIA-Group/BioSPPy). Additionally, we applied sequential forward feature selection (SFFS) in order to preserve only the most informative features, and save time and computational power of the machine learning algorithm to be applied in the next step. All the presented methods were implemented in Python and made available as open source software https://github.com/PIA-Group/BioSPPy.

### 3.2. Decision Fusion

In contrast to FF, in DF, from each sensor signal, a feature vector is extracted and used independently to train and learn a classifier, so that each modality returns a set of predicted labels. Hence, with *k* modalities, *k* classifiers will be created returning *k* predictions per sample. The returned predictions are then combined to yield a final result, in our case, via a weighted majority voting system. In this voting system, the ensemble decides on the class that receives the highest number of votes taking into account all sensor modalities, and a weight (*W*) parameter per modality to give the more competent classifiers a greater power for the final decision. The weights were chosen for each modality according to the classifier accuracy on the validation set. In case of a draw in the class prediction, the selection is random.

### 3.3. Classifier

To perform the classification seven SL classifiers were tested: K-Nearest Neighbour (k-NN); Decision Tree (DT); Random Forest (RF); Support Vector Machines (SVM); AdaBoost (AB); Gaussian Naive Bayes (GNB); and Quadratic Discriminant Analysis (QDA). For more detail regarding these classifiers, the author refers the reader to [48] and references therein.

A comprehensive study of these classifiers performance and parameter tunning was performed using 4-fold Cross Validation (CV) to ensure a meaningful validation and avoiding overfitting. The value of 4 was selected to optimise the number of iterations and the homogeneity in number of the classes in the training and test set, since some of the datasets used were highly imbalanced. The best performing classifier was chosen using Leave-One-Subject-Out (LOSO) to be incorporated into the FF and DF frameworks.

To obtain a measurable evaluation of the model performance, the following metrics are computed: Accuracy—$\frac{TP+TN}{TP+TN+FP+FN}$; Precision—$\frac{TP}{TP+FP}$; Recall—$\frac{TP}{TP+FN}$; F1-score—the harmonic mean of precision and recall [49]. Nomenclature: TP—True Positive; FP—False Positive; FP—False Positive; FN—False negative.

## 4. Experimental Results

In this section, we start by introducing the datasets used in this paper, followed by an analysis and classification performance comparison of the FF and DF approaches.

### 4.1. Datasets

In the scope of our work we used five publicly available datasets for ER, commonly used in previous work for benchmarking:

1. **IT Multimodal Dataset for Emotion Recognition (ITMDER)** [7]: contains the physiological signals of interest to our work (EDA, RESP, ECG, and BVP) of 18 individuals using two devices based on the BITalino system [50,51] (one placed on the arm and the other on the chest of the participants), collected while the subjects watched seven VR videos to elicit the emotions: Boredom, Joyfulness, Panic/Fear, Interest, Anger, Sadness and Relaxation. The ground-truth annotations were obtained by the subjects self-report per video using the Self-Assessment Manikin (SAM), in the Valence-Arousal space. For more information regarding the dataset, the authors refer the reader to [7].
2. **Multimodal Dataset for Wearable Stress and Affect Detection (WESAD) [6]:** contains EDA, ECG, BVP, and RESP sensors data collected from 15 participants using a chest- and a wrist-worn device: a RespiBAN Professional (biosignalsplux.com/index.php/respiban-professional) and an Empatica E4 (empatica.com/en-eu/research/e4) under 4 main conditions: Baseline (reading neutral magazines); Amusement (funny video clips); Stress (Trier Social Stress Test (TSST) consisting of public speaking and a mental arithmetic task); and lastly, meditation. The annotations were obtained using 4 self-reports: PANAS; SAM in Valence-Arousal space; State-Trait Anxiety Inventory (STAI); and Short Stress State Questionnaire (SSSQ). For more information regarding the dataset, the authors refer the reader to [6].
3. **A dataset for Emotion Analysis using Physiological Signals (DEAP) [8]:** contains EEG and peripheral (EDA, BVP, and RESP) physiological data from 32 participants, recorded as each watched 40 one-minute-long excerpts of music videos. The participants rated each video in terms of the levels of Arousal, Valence, like/dislike, dominance and familiarity. For more information regarding the dataset, the authors refer the reader to [8].
4. **Multimodal dataset for Affect Recognition and Implicit Tagging (MAHNOB-HCI) [52]:** contains face videos, audio signals, eye gaze data, and peripheral physiological data (EDA, ECG, RESP) of 27 participants watching 20 emotional videos, self-reported in Arousal, Valence, dominance, predictability, and additional emotional keywords. For more information regarding the dataset, the authors refer the reader to [52].
5. **Eight-Emotion Sentics Data (EESD) [9]:** contains physiological data (EMG, BVP, EDA, and RESP) from an actress during deliberate emotional expressions of Neutral, Anger, Hate, Grief, Platonic Love, Romantic Love, Joy, and Reverence. For more information regarding the dataset, the authors refer the reader to [9].

Table 1 shows a summary of the datasets used in this paper, highlighting their main characteristics. One should notice that the datasets are heavily imbalanced.

### 4.2. Signal Pre-Processing

The raw data recorded from the sensors usually shows a low signal-to-noise ratio, thus, it is generally necessary to pre-process the data, namely filtering to remove motion artefacts, outliers, and further noise. Additionally, since different modalities were acquired, different filtering specifications are required according to each sensor modality. Considering what is typically found in the state-of-the-art [11], the filtering for which each modality was performed as follows:

- **Electrocardiography (ECG):** Finite impulse response (FIR) band-pass filter of order 300 and 3–45 Hz cut-off frequency.
- **Electrodermal Activity (EDA):** Butterworth low-pass pass filter of order 4 and 1 Hz cut-off frequency.
- **Respiration (RESP):** Butterworth band-pass filter of order 2 and 0.1–0.35 Hz cut-off frequency.
- **Blood Volume Pulse (BVP):** Butterworth band-pass filter of order 4 and 1–8 Hz cut-off frequency.

After noise removal, the data was segmented into 40 s sliding windows with 75% overlap. Lastly, the data was normalised per user, by subtracting the mean and dividing by the standard deviation, to values between 0–1 to remove subjective bias.

### 4.3. Supervised Learning Using Single Modality Classifiers

The ER classification is performed with a classifier tuned for Arousal and another for Valence. Table 2 presents the experimental results for the SL techniques.

As it can be seen, for the ITMDER dataset, the state-of-the-art results [7] were available for each sensor modality, which we display and, overall our methodology was able to achieve superior results. Additionally, altogether, we observe higher accuracy values in the Valence dimension compared to the Arousal scale. Thirdly, for the WESAD dataset, the F1-score drops significantly to 0.0, compared to the Accuracy score value. The F1-score low value derives from the fact, that the class labels were largely unbalanced, with some of the test sets having none of one of the labels. To conclude, overall, all the sensors modalities display competitive results with no individual sensor modality standing out as the optimal for ER.

We present the classifiers used per sensor modality and class dimension in Table 3. Additionally, the features obtained using the forward feature selection algorithm are displayed in Table 4 and Table 5, for the Arousal and Valence dimensions, respectively. As shown, they explore similar correlated aspects in each modality.

Both the presented classifiers and features were selected via a 4-fold CV, to be used for the SL evaluation and for the DF algorithm, which is detailed in the next section. Hence, no classifier was generally able to emerge as the optimal for ER on the aforementioned axis. Lastly, concerning the features for each modality, we used 570, 373, 322, and 487 features respectively for the EDA, ECG, BVP, and RESP sensor data. However, such high dimension feature vector can be highly redundant and has many zero column features, therefore, we were able to reduce the feature vector without significant degradation of the classification performance.

**Figure A1:**
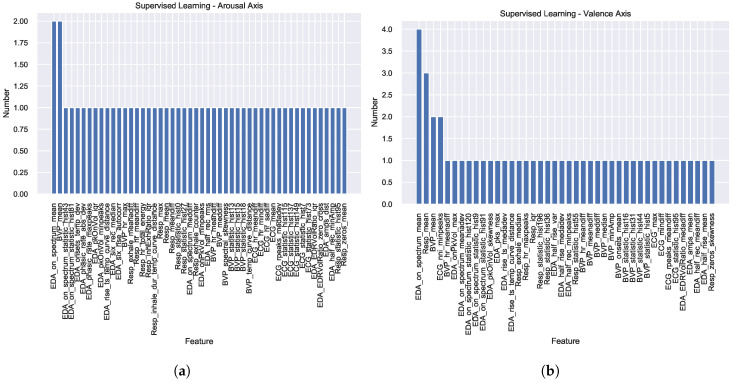
Histogram combining the features used in the SL (Supervised Learning) methodologies in all the datasets for the Arousal and Valence axis in (**a**,**b**), respectively. For information regarding the features the authors refer the reader to (https://github.com/PIA-Group/BioSPPy).

Figure A1 in Appendix A displays two histograms merging the features used in the SL methodologies in all the datasets for the Arousal and Valence axis, respectively. The figure shows that most features are selected via the SFFS methodology, specifically for each dataset (a value of 1 means that the features were selected in just one dataset). The features EDA onsets spectrum mean value, and BVP signal mean are selected in 2 datasets for the Arousal axis; while, the features EDA onsets spectrum mean value (in 4), RESP signal mean (in 2), BVP (in 2) signal mean, and ECG NNI (NN intervals) minimum peaks value, are repeated for the Valence axis.

### 4.4. Decision Fusion vs. Feature Fusion

In the current sub-section we present the experimental results for the DF and FF methodologies. Table 6 shows the experimental results in terms of Accuracy and F1-score for the Arousal and Valence dimensions in the 5 studied datasets, along with some state-of-the-art results. As it can be seen, once gain, both of our techniques outperform the results obtained for ITMDER [7], with more expression in the Valence dimension. Similarly for the DEAP dataset [8], where only for the Valence axis in terms of Accuracy we did not succeed, attaining, however, competitive results, and surpassing in terms of F1-score.

On the other hand, with the MAHNOB-HCI dataset [53], our proposal does not attain the literature results. For the EESD and the WESAD datasets, no state-of-the-art results are presented since it is yet, to the best of our knowledge, to be applied to ER. Thus, we denote as an un-explored annotation dimension which we evaluate in the present paper. Secondly, when comparing DF with FF, the former surpasses the latter for the EESD dataset in both the Arousal and Valence scale. For the remaining datasets, very competitive results are reached on both techniques. Regarding the computational time, FF is more competitive than DF, with an average execution time two orders of magnitude lower comparatively to DF (Language: Python 3.7.4; Memory: 16 GB 2133 MHz LPDDR3; Processor: 2.9 GHz Intel Core i7 quadruple core).

Table 7 presents the classifiers used per dataset and sensor modality for the Arousal and Valence dimension in the FF methodology.

The experimental results show that the selection was: 2 QDA; 1 SVM; 1 GNB; 1 DT (for the Arousal scale); and 2 RF; 1 SVM; 1 GNB; and 1 QDA (for the Valence scale). These results exhibit once again that, as for the SL techniques, no particular type of classifier was globally selected for all the datasets. Additionally, Table 8 displays the features used per dataset and sensor modality for the Arousal and Valence dimension in the FF methodology.

Results also showed that, similarly to the SL methodology, most features are specific per to a given dataset, with zero features being selected through the SFFS in common in all the datasets feature selection step.

In summary, this paper explored the datasets in new emotion dimensions and evaluation metrics yet to be reported in the literature, and attained similar or competitive results comparatively to the available state-of-the-art. The experimental results showed that between FF and DF using SL, very similar results are attained, and the best performing methodology is highly dependent on the dataset. These results are possibly due to the features being different for each dataset and sensor modality. In the SL classifier results, the best performing sensor modality is uncertain. While the DF methodology displayed the higher computation and time complexity. Therefore, considering these points, we select the FF methodology as the best modality fusion option since, with a single classifier, and pre-selected features, high results are reached with low processing time and computational complexity.

## 5. Conclusions and Future Work

Over the past decade, the field of affective computing has grown, with many datasets being created [6,7,8,9,52], however, a consolidation is lacking concerning: (1) What are the ranges of the expected classification performance; (2) The definition of the best sensor modality, SL classifier and features per modality for ER; (3) Which is the best technique to deal with multimodality and their limitations (FF or DF); (4) Selection of the classification model. Therefore, in this work, we studied the recognition of low/high emotional response in two dimensions: Arousal and Valence, for five publicly available datasets commonly found in literature. For this, we focus on physiological data sources easily measured from pervasive wearable technology, namely ECG, EDA, RESP and BVP data. Then, to deal with the multimodality, we analyse two techniques: FF and DF.

We extend the state-of-the-art with: (1) Benchmarking the ER classification performance for SL, FF and DF in a systematic way; (2) Summarising the accuracy and F1-score (important due to the imbalanced nature of the datasets); (3) Comprehensive study of SL classifiers and extended feature set for each modality; (4) Systematic analysis of multimodal classification in DF and FF approaches. We were able to obtain superior or comparable results to those found in literature for the selected datasets. Experimental results showed that FF is the most competitive technique.

For future work, we identified the following research lines: (1) Acquisition of additional data for the development of a subject-dependent model, since emotions are highly subject-dependent resulting, according to literature [11], in a higher classification performance; (2) Grouping the users by clusters of response might provide a look into sub-groups of personalities, a further parameter that must be taken into consideration when characterising emotion; (3) As stated in Section 4.3 we used a SFFS methodology to select the best feature set to use in all our tested techniques, however, it is not optimal, so the classification results using additional feature selection techniques should be tested; (4) Lastly, our work is highly conditioned on the extracted features, while lately, higher focus has been made to Deep Learning techniques, but in an approach where the feature extraction step is embedded in the neural network - ongoing work concerns the exploration and comparison of feature engineering and data representation learning approaches, with emphasis on performance and explainability aspects.

## Figures and Tables

**Table 1:** Summary of the datasets information on: classes; ratio of number (N°) of samples per class label shown between parenthesis—N° samples per class label / total number of samples, for the classes 0 and 1, shown between parenthesis; demographic Information (DI)—number of participants; ages (years old) ± standard deviation, and Female (F)-Male (M) subject distribution; device used for this paper; and sampling rate. Dataset nomenclature: ITMDER—IT Multimodal Dataset for Emotion Recognition; WESAD—Multimodal Dataset for Wearable Stress and Affect Detection; DEAP—A dataset for Emotion Analysis using Physiological Signals; MAHNOB-HCI—Multimodal dataset for Affect Recognition and Implicit Tagging; EESD—Eight-Emotion Sentics Data.

Dataset	Classes	N° of Samples per Class	DI	Device	Sampling Rate
ITMDER	Low-high Arousal/Valence	Arousal: 0.54 (0); 0.46 (1) Valence: 0.12 (0); 0.88 (1)	18 23 ± 3.7 10 (F) – 13 (M)	Chest strap and armband based on BITalino ^a^	1000
WESAD	Neutral, Stress, Amusement + 4 Questionnaires	Arousal: 0.86 (0); 0.14 (1) Valence: 0.07 (0); 0.93 (1)	15 27.5 ± 2.4 3 (F) – 12 (M)	RespiBAN Professional ^b^, Empatica E4	ECG and RESP: 700; EDA: 4; BVP: 64
DEAP	Arousal, Valence, Like/dislike, Dominance and Familiarity	Arousal: 0.41 (0); 0.59 (1) Valence: 0.43 (0); 0.57 (1)	32 16 (F) – 16 (M) 19 – 37	Biosemi Active II system ^c^	128
MAHNOB-HCI	Arousal, Valence, Dominance	Arousal: 0.48 (0); 0.52 (1) Valence: 0.47 (0); 0.53 (1)	27 26.06 ± 4.39 17(F) – 13(M)	Biosemi Active II system	256
EESD	Neutral, Anger, Hate, Grief, Platonic love, Romantic Love, Joy, and Reverence	Arousal: 0.5 (0); 0.5 (1) Valence: 0.5 (0); 0.5 (1)	1 1 (F)	Thought Technologies ProComp prototype ^d^	256

^a^https://bitalino.com/en/; ^b^https://biosignalsplux.com; ^c^https://www.biosemi.com; ^d^http://thoughttechnology.com/index.php/procomp-infiniti-343.html.

**Table 2:** Experimental results in terms of the classifier’s Accuracy (1st row) and F1-score (2nd row) in %. All listed values are obtained using Leave-One-Subject-Out (LOSO). Nomenclature: SOA—State-of-the-art results; EDA H, EDA F—EDA obtained on a device placed on the hand and finger, respectively. The SOA column contains the results found in the literature [7]. The best results are shown in bold.

	ITMDER				WESAD		DEAP		MAHNOB-HCI		EESD	
	**Arousal**	**SOA**	**Valence**	**SOA**	**Arousal**	**Valence**	**Arousal**	**Valence**	**Arousal**	**Valence**	**Arousal**	**Valence**
**EDA**	59.65 ± 13.46	0.572	89.26 ± 17.3	0.721	85.78 ± 16.55	92.86 ± 11.96	58.91 ± 15.21	56.56 ± 9.07	50.61 ± 21.84	56.43 ± 34.84	59.38 ± 16.24	**68.75 ± 18.75**
**H**	40.74 ± 26.0		93.2 ± 12.37		0.0 ± 0.0	95.86 ± 6.99	72.91 ± 12.92	71.83 ± 7.42	47.53 ± 31.47	**64.63 ± 34.57**	56.82 ± 20.8	**66.71 ± 23.1**
**EDA**	56.03 ± 11.0	0.572	90.91 ± 11.29	0.721						
**F**	45.67 ± 20.01		91.24 ± 18.75							
**ECG**	**68.33 ± 5.58**	0.656	89.26 ± 17.3	0.7	85.75 ± 16.61	92.86 ± 11.96			49.36 ± 37.5	**59.15 ± 24.5**		
	**58.79 ± 21.54**		93.2 ± 12.37		0.0 ± 0.0	95.86 ± 6.99			53.0 ± 39.62	56.58 ± 32.61		
**BVP**	58.44 ± 12.69	0.660	89.35 ± 17.23	0.695	85.78 ± 16.55	**94.39 ± 9.98**	58.88 ± 15.19	56.56 ± 9.07			67.5 ± 13.35	66.25 ± 16.35
	45.91 ± 25.24		93.25 ± 12.34		0.0 ± 0.0	**96.68 ± 6.01**	72.9 ± 12.91	71.83 ± 7.42			66.98 ± 15.95	64.49 ± 22.07
**RESP**	62.37 ± 16.83	0.585	89.26 ± 17.3	0.629	85.78 ± 16.55	92.86 ± 11.96	58.83 ± 14.78	56.56 ± 9.07	50.62 ± 21.25	46.57 ± 20.67	**72.5 ± 12.87**	67.5 ± 10.0
	51.79 ± 23.16		93.2 ± 12.37		0.0 ± 0.0	95.86 ± 6.99	72.6 ± 12.74	71.83 ± 7.42	44.28 ± 31.66	48.27 ± 28.44	**70.12 ± 15.72**	57.92 ± 15.12

**Table 3:** Classifier used per dataset and sensor modality for the Arousal and Valence dimensions respectively used in the SL and DF methodologies, obtained using 4-fold CV. Nomenclature: K-Nearest Neighbour (k-NN); Decision Tree (DT); Random Forest (RF); Support Vector Machines (SVM); Gaussian Naive Bayes (GNB); and Quadratic Discriminant Analysis (QDA).

	ITMDER		WESAD		DEAP		MAHNOB-HCI		EESD	
	**Arousal**	**Valence**	**Arousal**	**Valence**	**Arousal**	**Valence**	**Arousal**	**Valence**	**Arousal**	**Valence**
**EDA Hand**	DT	RF	RF	RF	SVM	SVM	AdaBoost	SVM	AdaBoost	AdaBoost
**EDA Finger**	AdaBoost	QDA								
**ECG**	AdaBoost	RF	QDA	RF			RF	AdaBoost		
**BVP**	QDA	RF	AdaBoost	RF	RF	RF			AdaBoost	AdaBoost
**Resp**	AdaBoost	RF	RF	RF	AdaBoost	RF	QDA	AdaBoost	AdaBoost	QDA

**Table 4:** Features used per dataset and sensor modality for the Arousal dimension in the SL and DF methodologies, obtained using 4-fold CV.

	ITMDER	WESAD	DEAP	MAHNOB-HCI	EESD
**EDA** **Hand**	peaksOnVol_minpeaks EDRVolRatio_iqr onsets_temp_dev	EDA_onsets_spectrum_mean	onsets_spectrum_mean	half_rec_minAmp half_rec_rms amplitude_dist onsets_spectrum_statistic_hist43 rise_ts_temp_curve_distance phasic_rate_maxpeaks onsets_spectrum_meddiff EDRVolRatio_zero_cross	phasic_rate_abs_dev onsetspeaksVol_minpeaks
**EDA** **Finger**	onsets_spectrum_statistic_hist81 peaksOnVol_iqr six_rise_autocorr				
**ECG**	statistic_hist73, statistic_hist115 hr_sadiff statistic_hist7 statistic_hist137	mean rpeaks_medadev hr_meandiff		hr_mindiff	
**BVP**	hr_max hr_meandiff	mean	mean		spectral_skewness temp_curve_distance statistic_hist18 statistic_hist13 statistic_hist15 meddiff
**RESP**	exhale_counter inhExhRatio_iqr	statistic_hist0	mean	hr_total_energy meandiff statistic_hist95 inhale_dur_temp_curve_distance statistic_hist27 hr_meandiff	exhale_meanadiff max, zeros_mean

**Table 5:** Features used per dataset and sensor modality for the Valence dimension in the SL and DF methodology, obtained using 4-fold CV.

	ITMDER	WESAD	DEAP	MAHNOB-HCI	EESD
**EDA Hand**	onsets_spectrum_mean rise_ts_temp_curve_distance rise_ts_medadev	onsets_spectrum_mean	onsets_spectrum_mean	onsets_spectrum_mean	amplitude_mean onsets_spectrum_meanadev half_rise_medadev onsets_spectrum_statistic_hist9 EDRVolRatio_medadiff half_rec_minpeaks
**EDA Finger**	onset_peaks_Vol_max half_rise_mean, peaks_max onsets_spectrum_statistic_hist120 half_rec_meandiff onsets_spectrum_statistic_hist91 half_rise_var peaks_Onset_Vol_skewness				
**ECG**	nni_minpeaks	nni_minpeaks statistic_hist95		rpeaks_meandiff max mindiff	
**BVP**	statistic_hist44 meanadiff hr_meanadiff onsets_mean hr_meandiff	median minAmp	mean		mean statistic_hist16 statistic_hist5 statistic_hist31 meddiff
**Resp**	mean exhale_median statistic_hist196	mean	mean	hr_maxpeaks statistic_hist55 zeros_skewness statistic_hist36	iqr

**Table 6:** Experimental results for the FF and DF methodologies in terms of Accuracy (A) and F1-score (F1), and time (T) in seconds, per dataset for the Arousal dimension in the FF methodology. Results obtained using LOSO. The SOA column contains the results found in the literature (ITMDER [7], DEAP [8], MAHNOB-HCI [53]). The best results are shown in bold.

	ITMDER				WESAD		DEAP				MAHNOB-HCI				EESD	
	**Arousal**	**SOA**	**Valence**	**SOA**	**Arousal**	**Valence**	**Arousal**	**SOA**	**Valence**	**SOA**	**Arousal**	**SOA**	**Valence**	**SOA**	**Arousal**	**Valence**
**DF**																
**A**	66.7 ± 9.0	58.1	89.3 ± 17.3	57.12	85.8 ± 16.5	92.9 ± 12.0	58.9 ± 15.2		56.6 ± 9.1		54.7 ± 13.3		58.1 ± 6.1		**75.0 ± 14.8**	**75.6 ± 17.9**
**F1**	**50.9 ± 23.5**		93.2 ± 12.4		0.0 ± 0.0	95.9 ± 7.0	72.9 ± 12.9		71.8 ± 7.4		63.8 ± 15.8		68.1 ± 8.9		**73.4 ± 16.4**	**72.4 ± 22.5**
**T**	1.5 ± 0.0		1.35 ± 0.0		2.04 ± 0.0	2.0 ± 0.0	1.58 ± 0.0		1.73 ± 0.0		1.1 ± 0.0		1.35 ± 0.0		0.6 ± 0.0	0.7 ± 0.0
**FF**																
**A**	**87.6 ± 16.7**		89.26 ± 17.3		**87.6 ± 16.7**	92.9 ± 12.0	60.0 ± 13.9	57.0	56.9 ± 8.2	62.7	55.2 ± 15.4	64.2 57 55 ± 3.9	56.0 ± 10.2	68.7 62.7 ± 3.9 57.5	60.0 ± 18.4	68.7 ± 22.2
**F1**	19.4 ± 34.4		93.2 ± 12.4		19.4 ± 34.4	95.9 ± 7.0	67.3 ± 23.8	53.3	70.7 ± 7.6	60.8	67.5 ± 16.6		59.0 ± 15.1		56.7 ± 22.5	67.7 ± 24.7
**T**	0.02 ± 0.0		0.02 ± 0.0		0.02 ± 0.0	0.07 ± 0.01	0.02 ± 0.01		0.02 ± 0.0		0.01 ± 0.0		0.01 ± 0.0		0.0 ± 0.0	0.01 ± 0.0

**Table 7:** Classifier used per dataset and sensor modality for the Arousal and Valence dimension in the FF methodology. Results obtained using 4-fold CV.

	ITMDER		WESAD		DEAP		MAHNOB-HCI		EESD	
	Arousal	Valence	Arousal	Valence	Arousal	Valence	Arousal	Valence	Arousal	Valence
**Classifier**	SVM	RF	QDA	SVM	QDA	GNB	GNB	QDA	DT	RF

**Table 8:** Features used per dataset and sensor modality for the Arousal and Valence dimension in the FF methodology. Results obtained using 4-fold CV.

ITMDER	WESAD	DEAP	MAHNOB-HCI	EESD
**Arousal**				
EDA_H_onsets_spectrum_mean	BVP_median ECG_min Resp_statistic_hist64	Resp_zeros_sadiff BVP_statistic_hist29 EDA_phasic_rate_total_energy EDA_rise_ts_mindiff Resp_statistic_hist25	Resp_inhExhRatio_maxpeaks EDA_phasic_rate_iqr Resp_inhExhRatio_zero_cross Resp_inhExhRatio_skewness ECG_rpeaks_meanadiff ECG_minpeaks Resp_meandiff EDA_onsets_spectrum_minAmp EDA_onsets_spectrum_statistic_hist22 ECG_hr_dist EDA_onsets_spectrum_statistic_hist62	Resp_exhale_max EDA_amplitude_kurtosis
**Valence**				
EDA_H_peaksOnVol_minAmp BVP_mean EDA_F_EDRVolRatio_total_energy EDA_H_onsets_spectrum_statistic_hist112	BVP_median ECG_dist ECG_zero_cross ECG_statistic_hist143	Resp_statistic_hist60 EDA_half_rise_dist BVP_statistic_hist10 BVP_statistic_hist39 EDA_half_rise_temp_curve_distance BVP_hr_maxAmp	ECG_meanadiff EDA_rise_ts_meandiff Resp_inhale_dur_dist EDA_onsets_spectrum_statistic_hist5	EDA_amplitude_mean BVP_statistic_hist35 Resp_rms Resp_zeros_meandiff EDA_onsets_spectrum_statistic_hist22

## References

[1] Greenberg L.S., Safran J. (1987). Emotion, Cognition, and Action. Theoretical Foundations of Behavior Therapy.

[2] Shu L., Xie J., Yang M., Li Z., Li Z., Liao D., Xu X., Yang X. (2018). A Review of Emotion Recognition Using Physiological Signals. Sensors.

[3] Paul E. (1992). An argument for basic emotions. Cogn. Emot..

[4] Damasio A.R. (1994). Descartes’ Error: Emotion, Reason, and the Human Brain.

[5] Lang P.J. (1995). The emotion probe: Studies of motivation and attention. Am. Psychol..

[6] Schmidt P., Reiss A., Duerichen R., Marberger C., Van Laerhoven K. Introducing WESAD, a Multimodal Dataset for Wearable Stress and Affect Detection. Proceedings of the International Conference on Multimodal Interaction.

[7] Pinto J. (2019). Exploring Physiological Multimodality for Emotional Assessment. Master’s Thesis.

[8] Koelstra S., Muhl C., Soleymani M., Lee J., Yazdani A., Ebrahimi T., Pun T., Nijholt A., Patras I. (2012). DEAP: A Database for Emotion Analysis using Physiological Signals. IEEE Trans. Affect. Comput..

[9] Picard R.W., Vyzas E., Healey J. (2001). Toward machine emotional intelligence: Analysis of affective physiological state. IEEE Trans. Pattern Anal. Mach. Intell..

[10] Schmidt P., Reiss A., Duerichen R., Laerhoven K.V. (2018). Wearable affect and stress recognition: A review. arXiv.

[11] Bota P.J., Wang C., Fred A.L.N., Plácido da Silva H. (2019). A Review, Current Challenges, and Future Possibilities on Emotion Recognition Using Machine Learning and Physiological Signals. IEEE Access.

[12] Liu C., Rani P., Sarkar N. An empirical study of machine learning techniques for affect recognition in human-robot interaction. Proceedings of the International Conference on Intelligent Robots and Systems.

[13] Kim S.M., Valitutti A., Calvo R.A. Evaluation of Unsupervised Emotion Models to Textual Affect Recognition. Proceedings of the NAAL HLT Workshop on Computational Approaches to Analysis and Generation of Emotion in Text.

[14] Zhang Z., Han J., Deng J., Xu X., Ringeval F., Schuller B. (2018). Leveraging Unlabeled Data for Emotion Recognition with Enhanced Collaborative Semi-Supervised Learning. IEEE Access.

[15] Alhagry S., Fahmy A.A., El-Khoribi R.A. (2017). Emotion Recognition based on EEG using LSTM Recurrent Neural Network. Int. J. Adv. Comput. Sci. Appl..

[16] Zhang J., Chen M., Hu S., Cao Y., Kozma R. PNN for EEG-based Emotion Recognition. Proceedings of the International Conference on Systems, Man, and Cybernetics.

[17] Salari S., Ansarian A., Atrianfar H. Robust emotion classification using neural network models. Proceedings of the Iranian Joint Congress on Fuzzy and Intelligent Systems.

[18] Vanny M., Park S.M., Ko K.E., Sim K.B., Kim J.H., Matson E.T., Myung H., Xu P. (2013). Analysis of Physiological Signals for Emotion Recognition Based on Support Vector Machine. Robot Intelligence Technology and Applications 2012.

[19] Cheng B. (2012). Emotion Recognition from Physiological Signals Using Support Vector Machine.

[20] He C., Yao Y.J., Ye X.S. (2017). An Emotion Recognition System Based on Physiological Signals Obtained by Wearable Sensors.

[21] Meftah I.T., Le Thanh N., Ben Amar C., Huang T., Zeng Z., Li C., Leung C.S. (2012). Emotion Recognition Using KNN Classification for User Modeling and Sharing of Affect States. Proceedings of the Neural Information Processing.

[22] Li M., Xu H., Liu X., Lu S. (2018). Emotion recognition from multichannel EEG signals using K-nearest neighbor classification. Technol. Health Care.

[23] Kolodyazhniy V., Kreibig S.D., Gross J.J., Roth W.T., Wilhelm F.H. (2011). An affective computing approach to physiological emotion specificity: Toward subject-independent and stimulus-independent classification of film-induced emotions. Psychophysiology.

[24] Zhang X., Xu C., Xue W., Hu J., He Y., Gao M. (2018). Emotion Recognition Based on Multichannel Physiological Signals with Comprehensive Nonlinear Processing. Sensors.

[25] Gong P., Ma H.T., Wang Y. Emotion recognition based on the multiple physiological signals. Proceedings of the International Conference on Real-time Computing and Robotics.

[26] Ayata D., Yaslan Y., Kamasak M.E. (2020). Emotion Recognition from Multimodal Physiological Signals for Emotion Aware Healthcare Systems. J. Med. Biol. Eng..

[27] Chen J., Hu B., Wang Y., Moore P., Dai Y., Feng L., Ding Z. (2017). Subject-independent emotion recognition based on physiological signals: A three-stage decision method. BMC Med. Informatics Decis. Mak..

[28] Damaševičius R., Zhuang N., Zeng Y., Tong L., Zhang C., Zhang H., Yan B. (2017). Emotion Recognition from EEG Signals Using Multidimensional Information in EMD Domain. BioMed Res. Int..

[29] Lahane P., Sangaiah A.K. (2015). An Approach to EEG Based Emotion Recognition and Classification Using Kernel Density Estimation. Procedia Comput. Sci..

[30] Qing C., Qiao R., Xu X., Cheng Y. (2019). Interpretable Emotion Recognition Using EEG Signals. IEEE Access.

[31] Xianhai G. (2011). Study of Emotion Recognition Based on Electrocardiogram and RBF neural network. Procedia Eng..

[32] Xiefeng C., Wang Y., Dai S., Zhao P., Liu Q. (2019). Heart sound signals can be used for emotion recognition. Sci. Rep..

[33] Dissanayake T., Rajapaksha Y., Ragel R., Nawinne I. (2019). An Ensemble Learning Approach for Electrocardiogram Sensor Based Human Emotion Recognition. Sensors.

[34] Shukla J., Barreda-Angeles M., Oliver J., Nandi G.C., Puig D. (2019). Feature Extraction and Selection for Emotion Recognition from Electrodermal Activity. IEEE Trans. Affect. Comput..

[35] Udovičić G., Ðerek J., Russo M., Sikora M. Wearable Emotion Recognition System Based on GSR and PPG Signals. Proceedings of the 2nd International Workshop on Multimedia for Personal Health and Health Care.

[36] Liu M., Fan D., Zhang X., Gong X. Human Emotion Recognition Based on Galvanic Skin Response Signal Feature Selection and SVM. Proceedings of the 2016 International Conference on Smart City and Systems Engineering.

[37] Wei W., Jia Q., Yongli F., Chen G. (2018). Emotion Recognition Based on Weighted Fusion Strategy of Multichannel Physiological Signals. Comput. Intell. Neurosci..

[38] Chen J., Hu B., Xu L., Moore P., Su Y. Feature-level fusion of multimodal physiological signals for emotion recognition. Proceedings of the International Conference on Bioinformatics and Biomedicine (BIBM).

[39] Canento F., Fred A., Silva H., Gamboa H., Lourenço A. Multimodal biosignal sensor data handling for emotion recognition. Proceedings of the 2011 IEEE Sensors Conference.

[40] Xie J., Xu X., Shu L. WT Feature Based Emotion Recognition from Multi-channel Physiological Signals with Decision Fusion. Proceedings of the Asian Conference on Affective Computing and Intelligent Interaction.

[41] Subramanian R., Wache J., Abadi M.K., Vieriu R.L., Winkler S., Sebe N. (2018). ASCERTAIN: Emotion and Personality Recognition Using Commercial Sensors. IEEE Trans. Affect. Comput..

[42] Aguileta A.A., Brena R.F., Mayora O., Molino-Minero-Re E., Trejo L.A. (2019). Multi-Sensor Fusion for Activity Recognition—A Survey. Sensors.

[43] Egger M., Ley M., Hanke S. (2019). Emotion Recognition from Physiological Signal Analysis: A Review. Electron. Notes Theor. Comput. Sci..

[44] Doma V., Pirouz M. (2020). A comparative analysis of machine learning methods for emotion recognition using EEG and peripheral physiological signals. J. Big Data.

[45] Dzedzickis A., Kaklauskas A., Bucinskas V. (2020). Human Emotion Recognition: Review of Sensors and Methods. Sensors.

[46] Marechal C., Mikołajewski D., Tyburek K., Prokopowicz P., Bougueroua L., Ancourt C., Węgrzyn-Wolska K. (2019). High-Performance Modelling and Simulation for Big Data Applications: Selected Results of the COST Action IC1406 cHiPSet.

[47] Zhang J., Yin Z., Chen P., Nichele S. (2020). Emotion recognition using multi-modal data and machine learning techniques: A tutorial and review. Inf. Fusion.

[48] Duda R.O., Hart P.E., Stork D.G. (2000). Pattern Classification.

[49] Pedregosa F., Varoquaux G., Gramfort A., Michel V., Thirion B., Grisel O., Blondel M., Prettenhofer P., Weiss R., Dubourg V. (2011). Scikit-learn: Machine Learning in Python. J. Mach. Learn. Res..

[50] Da Silva H.P., Fred A., Martins R. (2014). Biosignals for Everyone. IEEE Pervasive Comput..

[51] Alves A.P., Plácido da Silva H., Lourenco A., Fred A. BITalino: A Biosignal Acquisition System based on Arduino. Proceedings of the International Conference on Biomedical Electronics and Devices (BIODEVICES).

[52] Soleymani M., Lichtenauer J., Pun T., Pantic M. (2012). A Multimodal Database for Affect Recognition and Implicit Tagging. IEEE Trans. Affect. Comput..

[53] Wiem M., Lachiri Z. (2017). Emotion Classification in Arousal Valence Model using MAHNOB-HCI Database. Int. J. Adv. Comput. Sci. Appl..

